# Chicken blood provides a suitable meal for the sand fly *Lutzomyia longipalpis *and does not inhibit *Leishmania *development in the gut

**DOI:** 10.1186/1756-3305-3-3

**Published:** 2010-01-11

**Authors:** Mauricio RV Sant'Anna, Alexandre Nascimento, Bruce Alexander, Erin Dilger, Reginaldo R Cavalcante, Hector M Diaz-Albiter, Paul A Bates, Rod J Dillon

**Affiliations:** 1 Liverpool School of Tropical Medicine, Pembroke Place, Liverpool, L3 5QA, UK; 2 Departamento de Parasitologia e Microbiologia, CCS, Universidade Federal do Piauí, Teresina, Piauí, Brazil; 3 Biomedical and Life Sciences, School of Health and Medicine, Lancaster University, UK; 4 Current address: Biological Sciences, University of Warwick, Coventry, CV4 7AL

## Abstract

**Background:**

The aim of this study was to address the role of chickens as bloodmeal sources for female *Lutzomyia longipalpis *and to test whether chicken blood is harmful to *Leishmania *parasite development within the sand flies. Bloodmeal ingestion, excretion of urate, reproduction, fecundity, as well as *Leishmania *infection and development were compared in sand flies fed on blood from chickens and different mammalian sources.

**Results:**

Large differences in haemoglobin and protein concentrations in whole blood (dog>human>rabbit> chicken) did not correlate with differences in bloodmeal protein concentrations (dog = chicken>human>rabbit). This indicated that *Lu. longipalpis *were able to concentrate bloodmeals taken from different hosts using prediuresis and this was confirmed by direct observation. Sand flies fed on chickens or dogs produced significantly more eggs than those fed on human blood. Female *Lu. longipalpis *retained significantly more urate inside their bodies when fed on chicken blood compared to those fed on rabbit blood. However, when the amounts of urate excreted after feeding were measured, sand flies fed on rabbit blood excreted significantly more than those fed on chicken blood. There was no difference in female longevity after feeding on avian or mammalian blood.

Sand flies infected via chicken blood produced *Leishmania mexicana *infections with a similar developmental pattern but higher overall parasite populations than sand flies infected via rabbit blood.

**Conclusions:**

The results of this study help to define the role that chickens play in the epidemiology of leishmaniasis. The present study using a *Lu. longipalpis*/*L. mexicana *model indicates that chickens are suitable hosts to support a *Lu. longipalpis *population and that chicken blood is likely to support the development of transmissible *Leishmania *infections in *Lu. longipalpis*.

## Background

The phlebotomine sand fly *Lutzomyia longipalpis *s.l. (Lutz & Neiva, 1912) (Diptera: Psychodidae) is the principal New World vector of *Leishmania (Leishmania) infantum *(syn. *chagasi*), aetiological agent of zoonotic visceral leishmaniasis (ZVL) [1]. Although originally associated with open, semi-arid areas such as NE Brazil, during the last two decades ZVL foci have appeared in many Brazilian cities where *Lu. longipalpis *has become established in the marginal neighbourhoods known as "*favelas*" [2,3]. As in other haematophagous Diptera, female sand flies take blood meals as protein sources for oocyte production and maturation [4]. Therefore, sand fly reproduction in urban environments depends on the availability of blood meal sources from synanthropic species of wild and domestic animals. The most medically important blood source is generally considered to be the domestic dog, an amplification host and reservoir for *L. infantum*, whereas man is a dead-end host for this parasite. In cities there are several other potential sources of blood, including wild animals such as opossums (*Didelphis albiventris*) and domestic livestock such as pigs, goats and chickens. The last of these is usually the most numerically important, many families keeping poultry for a variety of reasons [2].

As with other birds, chickens do not support *Leishmania *infections and appear to have no direct role in the transmission cycle of ZVL. Chickens do not harbour the infection possibly as a result of their higher natural body temperature of 41°C, or biological differences inherent within the chicken such as complement or nucleated RBCs [5,6]. Alexander et al. [2] postulated that ingestion of chicken blood by a vector carrying an established infection may have the effect of damaging the established parasites, thus temporarily making the insect refractory to *Leishmania*. Paradoxically, proximity of chickens was often cited as a risk factor for humans acquiring ZVL [7-10] and, therefore, any zooprophylactic action the birds may exert on transmission of *L. infantum *cannot be very effective. Several Brazilian control programmes have attempted to target chicken coops with insecticide spraying [2] and there are renewed efforts to develop pheromone traps in association with chickens [11]. However, previous studies comparing sand fly development following feeding on live animals have not examined avian hosts, perhaps because of the relative difficulty of anaesthetizing birds and the perception that they were irrelevant to *Leishmania *transmission.

Regarding nutritional value, chicken blood is potentially less suitable for sand fly development as it has a haematocrit value lower than that of other domestic animals [12]. Therefore, to obtain the same volume of red blood cells from an avian blood meal would require the insects to compensate and concentrate a chicken bloodmeal by prediuresis. However, while prediuresis (when feeding on mammals) has been observed in *Phlebotomus *species [13], it was reported to be absent in *Lu. longipalpis *[4]. Avian blood may also differ from mammalian blood in terms of sand fly digestive physiology, since the red blood cells of the former are nucleated. In particular urate excretion may be affected, a major product of DNA and protein catabolism as well as an important antioxidant in insects [14,15].

The aim of this study was to address the role of chickens as bloodmeal sources for *Lu. longipalpis *and to test whether chicken blood was harmful to *Leishmania *parasite development within the sand fly *Lu. longipalpis*.

## Methods

### Insects

A laboratory colony of *Lu. longipalpis *established from flies caught in Jacobina (Bahia-Brazil) and kept at the Liverpool School of Tropical Medicine was maintained using standard methods [16] and used in the protein, haemoglobin and urate assays. A second laboratory colony of *Lu. longipalpis *that originated from specimens collected in Teresina in the Brazilian state of Piauí was reared in the Núcleo de Entomologia do Piauí (NEPI) of the Universidade Federal do Piauí (UFPI) and used in live host feeding, oviposition and sand fly development experiments. All experiments were performed with 3-5 day old adult females.

### Haematological analysis of blood from chickens and mammalian hosts

Chicken and rabbit blood in Alsever's solution purchased from TCS Biosciences (Buckingham, UK), human blood obtained from the National Blood Service (Speke, Liverpool, UK) and dog blood purchased from Harlan Sera Lab Ltd (Leicestershire) were analysed using a Beckman Couter A^c ^T Hematology Analyzer/Counter.

### Protein assay

Total amounts of protein in different types of blood and in the midguts (4 hours post feed) of blood-fed *Lu. longipalpis *were quantified according to Bradford [17] and adapted to a 96-well plate assay. Briefly, 4 μL volumes of blood or gut homogenates prepared in 0.1 M Tris-HCl pH 7.5 were mixed with 196 μL of the BIORAD^® ^Protein assay reagent, diluted 1× in distilled water and absorbance measured in 96-well plates at 595 nm. Bovine serum albumin (BSA) was used as a standard.

### Haemoglobin assay on sand fly midgut homogenates

Haemoglobin was determined by colorimetry essentially as previously described [18]. Individual midguts of sand flies bloodfed on chicken, rabbit, human and dog blood were dissected 4 h after blood-feeding and transferred to 1.5 mL Eppendorf microfuge tubes containing 100 μL 0.15 mM NaCl. After homogenisation using a motorised mini-pestle and rapid centrifugation (1800 g for 30 s) to remove large debris, 20 μL volumes of sand fly midgut homogenate were mixed with 200 μL of Drabkin's reagent (Sigma) in the dark for 30 min. Next, 200 μL samples were transferred to 96-well microtitre plates and assayed at 540 nm. Human haemoglobin (Sigma) was used as standard.

### Observation of prediuresis

Individual 3 to 5 day old female *Lu. longipalpis *were given access to the forearm of two human volunteers and allowed to pursue normal bloodfeeding behaviour: short hopping flight and searching for a suitable feeding site. As soon as feeding had begun the fly was continuously observed under a binocular stereomicroscope at 40× magnification. The abdomen was seen to swell with ingestion of blood over a period of 3-5 minutes. Particular attention was paid to the tip of the abdomen noting the appearance of any drops of fluid during feeding.

### Uric acid assay

To measure urate in the whole body of *Lu. longipalpis *females a colorimetric assay was performed using the Amplex Red Uric Acid Assay kit (Invitrogen^®^). Insects were fed on chicken and rabbit blood and fully-engorged insects were collected at 12, 24, 48, 72 and 96 h after bloodfeeding. Sand flies were transferred to 1.5 mL Eppendorf tubes (two insects per vial) and homogenised in 130 μL of 0.1 M Tris-HCl pH 7.5 using a motorised mini-pestle. Following centrifugation at 13,500 rpm for 15 min, 50 μL of the homogenate was mixed with 50 μL of the Amplex Red Reagent (made up in 0.4 U/mL horseradish peroxidase, 0.4 U/mL uricase and 0.1 M Tris-HCl pH 7.5). Absorbance was measured at 490 nm after a 30 min incubation at 37°C. For urate measurements in excretions of *Lu. longipalpis *flies were fed on chicken and rabbit blood and stored alive individually inside microfuge tubes with pierced lids to allow ventilation. Tubes were placed inside sealed plastic boxes over humidified filter paper and adult females and excretions were homogenised, and urate collected by elution with 130 μL of 0.1 M Tris-HCl pH 7.5. Control blanks consisting of 50 μL 0.1 M Tris-HCl pH 7.5 and 50 μL of the sand fly homogenates were also read at 490 nm and subtracted from the experimental results.

### Sand fly oviposition and larval development

Four day old female sand flies were allowed to feed on chickens (n = 3), dogs (n = 3) or human volunteers. Chickens were restrained during feeding and dogs sedated with 2% xylazine at 1-3 mg/Kg body weight. All procedures involving animals were performed by a qualified veterinarian and in accordance with Brazilian government regulations. Time to engorge was estimated beginning after the mouthparts were inserted into the skin. Engorged sand flies were retained in the cages for a further 24 h before being transferred to individual plaster-lined (20 ml) oviposition pots. Each insect was kept in the pot and provided with a cotton wool pad moistened with 70% sugar solution until it laid eggs and died. Numbers of eggs (Christophers stage IV or V) retained within the body of the dead insects were also counted and added to the total laid by each individual. Eggs produced by blood-fed females were transferred to plaster-lined (100 ml) rearing pots, so that each contained an equal number of eggs laid on the same day. Development of progeny of females fed on dog and chicken blood was monitored until emergence of the last adult sand fly. The numbers of adult insects produced by each group and male/female sex ratios were recorded.

### *Leishmania *Infection

Cultured amastigotes of *Leishmania mexicana *(WHO reference strain MNYC/BZ/62/M379) were used for all infection work. Amastigotes were cultured, as previously detailed by Bates et al. [19] in Grace's culture medium (pH = 5.4) and enriched with 20% (v/v) foetal calf serum.

Approximately 600 newly emerged sand-flies were collected and separated into two groups five days prior to infection. The two groups of sand-flies were then given the infection by feeding upon blood inoculated with 2 × 10^6 ^*L. mexicana *cultured amastigotes per millilitre of blood [20]. The well-mixed, infected blood was presented to the sand-flies via a membrane feeding system secured within each cage, whereby a chick skin membrane was secured over the end of a glass feeding tube and then filled with 1.5 ml of the infected blood and heated to 35°C. One group of sand-flies was fed amastigotes with whole rabbit blood (control group) and the second group was concurrently fed amastigotes with whole chicken blood. Fully engorged females from each group were then removed to fresh cages. During and post-infection the flies were maintained at 24°C and 90% R.H., and given access to 70% sucrose solution to enhance the survival of the sand flies. Each experiment was replicated at least twice.

Ten fully engorged females were dissected from each group every 48 hours for eight days post-infection. The guts were homogenised in 50 μl of 0.15 mM NaCl and number of parasites per fly determined by counting using a haemocytometer. Five additional samples were selected at random from each experimental group every 48 hours for differential counting. 10 μl of midgut homogenate was placed onto a glass slide and Giemsa stained for estimation of the different morphological forms as described by Rogers et al. [18].

### Statistical analysis

The Anderson-Darling test was used to assess whether each variable was normally distributed. Those that showed normal distribution were compared by ANOVA, followed by independent t-tests. Those following non-normal distributions were compared using the Kruskal-Wallis, followed by *U *Mann-Whitney multiple comparison test. Differential counts were compared using χ^2^. Differences were considered to be significant at P ≤ 0.05.

## Results and Discussion

### Analysis of blood sources and ingestion by sand flies

**Figure 1:**
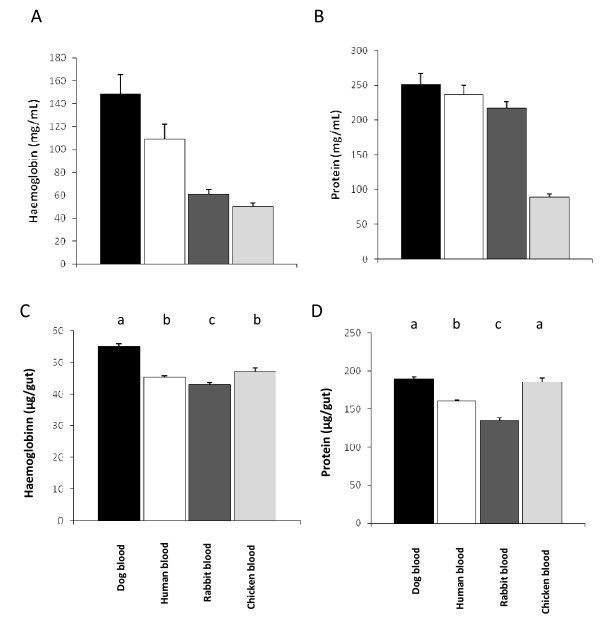
**Haemoglobin and protein content of whole blood and midguts of blood fed sand flies**. (A) Haemoglobin content of dog (n = 3), human (n = 5), rabbit (n = 14) and chicken (n = 6) blood. (B) Protein content of dog (n = 3), human (n = 4), rabbit (n = 8) and chicken (n = 6) blood. (C) Haemoglobin content of midgut lysates of *Lu. longipalpis *fed on dog (n = 80), human (n = 95), rabbit (n = 67) and chicken (n = 24) blood. Midguts were dissected 4 hours after bloodfeed. (D) Total protein content of midgut lysates of *Lu. longipalpis *fed on dog (n = 80), human (n = 95) rabbit (n = 48) and chicken blood (n = 24). Bars with different letters represent statistical significance at P ≤ 0.001 between blood sources, *(U *Mann-Whitney).

To investigate the influence that bloodmeal source might have on nutritional value to sand flies four different types of blood were examined: dog, human, rabbit and chicken. With regard to *Lutzomyia longipalpis *these represent the reservoir host for *Leishmania infantum *(dog), the human host, rabbit as a common laboratory blood source used for membrane feeding, and chickens as an important environmental blood source. Total protein and haemoglobin content were determined in dog, human, rabbit and chicken blood, along with a range of other haematological parameters (Additional file 1). This analysis revealed that both dog and human blood contained significantly higher amounts of haemoglobin than rabbit or chicken blood (P ≤ 0.024, Fig. 1A). The total protein content was less variable, being relatively consistent among the mammalian blood sources examined (Fig. 1B). However, chicken blood had less than half the total protein of any mammalian blood analysed (P ≤ 0.024) this was consistent with the low haematocrit and plasma protein concentration (not shown). Despite these variations, the volumes and haemoglobin contents for the erythrocytes themselves (MCV, MCH, MCHC) were as expected, for example, note the larger size of the (nucleated) avian erythrocytes.

Given the wide range of haemoglobin and protein contents in these blood sources, we examined the effect that this might have on bloodfeeding and subsequent digestion in *Lu. longipalpis*. Flies were fed by membrane on the four types of blood, fully engorged flies collected and the haemoglobin and total protein contents of sand fly midguts were determined at four hours post-feeding (Fig. 1C, D). Although the midguts of sand flies fed on dog blood showed higher haemoglobin levels, a difference that was statistically significant (P ≤ 0.001), no significant differences were seen between haemoglobin of human and chicken-fed midguts. Sand flies fed on rabbit blood showed the lowest haemoglobin concentration in their midguts (P ≤ 0.001) (Fig. 1C). A comparison of the total protein in the midguts of *Lu. longipalpis *fed on all four types of blood (Fig. 1D) showed no difference between dog and chicken fed sand flies. This suggested that the female sand fly was able to compensate for wide variations in haemoglobin and protein content of different blood sources. This is particularly relevant to the potential suitability of chickens as sources of a bloodmeal, since their blood has a haematocrit value, protein and haemoglobin content lower than that of mammalian blood (Additional File 1 Table A, [21]).

### Observation of prediuresis

One obvious explanation for the preceeding data is that the flies are practising prediuresis. This would have the effect of concentrating the red cell content of the bloodmeal and would act as an equalising factor when blood sources of differing composition are ingested. Various insects concentrate their bloodmeals by prediuresis [22] but the situation in sand flies was unclear. Concentration was not previously recorded in *Lu. longipalpis *[4], but was seen in the majority of *Phlebotomus argentipes *[23], while other *Phlebotomus *species fed on live hosts or force-fed by glass capillary excrete drops of fluid from the anus, concentrating their bloodmeals [13,24]. All flies (n = 20) in the present study were observed to be extruding small droplets of fluid from the anus during blood feeding. The droplets were observed to gradually increase in size and then rapidly disperse. The average number of droplets extruded (median) was 20 per fly (lower quartile = 6; upper quartile = 62) and the fluid extruded was yellowish in colour. These observations demonstrate that *Lu. longipalpis *is capable of prediuresis, and suggest it may be a frequent phenomenon during bloodfeeding for this sand fly species.

### Sand fly digestion

The data described indicate that variation in the nutritional quality of a bloodmeal source can be at least partially compensated by the action of prediuresis. However, there are other factors beyond the cellular content that could also affect the nutritional quality of a bloodmeal. One of the most obvious factors when comparing mammalian and avian blood is the presence of nucleated red blood cells in the latter. Chicken blood contains approximately 30 times more DNA than mammals [25]. During bloodmeal digestion, purines and protein are metabolised into urate, this being the main end-product of nitrogen metabolism in insects [26]. Urate is released into the haemolymph and absorbed by the malpighian tubules via a pH gradient to constitute a major excretory product [14].

**Figure 2:**
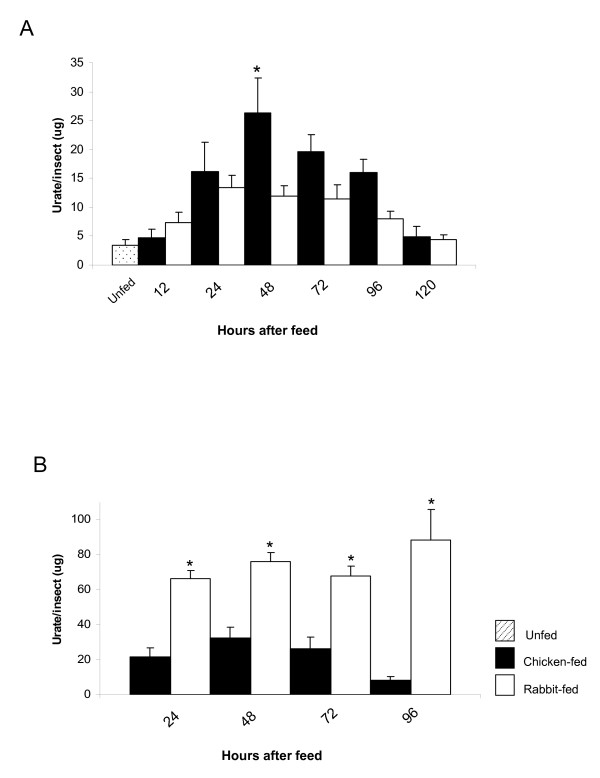
**Amount of urate in the whole body and excretions of sand flies fed on chicken and rabbit blood**. (A) Amount of urate in the whole bodies of individual *Lu. longipalpis *fed on chicken and rabbit blood. (B) Amount of urate in the whole body and excretions of individual *Lu. longipalpis *fed on chicken and rabbit blood. Results are presented as mean ± SEM of 4 independent experiments. Asterisks represent statistical significance at P ≤ 0.05 between chicken -fed and rabbit-fed sand flies.

Differences in urate production were measured and compared in flies that had membrane fed on either chicken or rabbit blood. Assays were performed on both whole *Lu. longipalpis *bodies and whole bodies plus excretory material in an attempt to estimate overall urate production for both types of blood. Urate content increased following bloodfeeding, reaching a peak at 24-48 hours (Fig. 2A). Flies contained more urate when fed on chicken blood compared to rabbit blood (P ≤ 0.05 at 48 h post-bloodmeal). However, when considering the total amount of urate (bodies + excretions), sand flies fed on rabbit blood consistently produced more urate than sand flies fed on chicken blood (P ≤ 0.05; Fig. 2B) for all time points measured.

Higher urate concentrations in the body may potentially lead to an increase in longevity of blood fed flies as urate is an important scavenger of free radicals produced as a consequence of haem production in bloodmeal-derived oxidative stress [14]. For example the urate-null rosy mutants of *Drosophila melanogaster*, defective for xanthine dehydrogenase the enzyme that generates urate, are sensitive to oxygen-derived stress [27] and RNAi silencing of xanthine dehydrogenase in *Lu. longipalpis *is detrimental to survival [15]. However, higher urate concentrations in the bodies of chicken fed flies were not reflected in greater longevity in comparison with flies fed on rabbit blood (Log Rank Mantel Cox test on sand fly survival monitored for 10 days; Additional File 2, Figure A). Presumably this was because sufficient urate was generated in rabbit blood-fed flies to satisfy their antioxidant needs.

### Sand fly feeding on live hosts

The effect of bloodfeeding from different live hosts on the feeding behaviour and reproductive success of *Lu. longipalpis *was investigated. Flies were given access to live restrained animal hosts or human volunteers and various parameters examined (Table 1). There was no significant difference between the percentages of sand flies that fed when offered one of the three hosts. The time taken to engorge on different hosts (in seconds) varied considerably within groups. However, the average feeding time to repletion was quickest on human hosts (320.1 ± 85.3), which was significantly different to both chickens and dogs (P ≤ 0.05).

**Table 1:** Effects of bloodfeeding on different live hosts on the reproduction of *Lu. longipalpis*.

	Chicken	Dog	Human
Proportion of flies feeding (%)	88.3	75.8	81.0
Time to engorge (s)	460.8 ± 218.2 (N = 20)	394.3 ± 129.8 (N = 20)	320.1 ± 85.3 (N = 20)*
Egg maturation time (days)	9.3 ± 1.7	9.1 ± 2.1	9.5 ± 2
Number of eggs	64.3 ± 2.2	64.3 ± 2.4	48 ± 1.7*
Post feeding survival (days)	9.8 ± 1.6	10 ± 1.8	10.5 ± 2
Post oviposition survival (days)	0.7 (52.9%)	0.8 (60.7%)	1.0 (61.7%)
Larval hatching time (days)	16.2 ± 2.5	16.2 ± 2.3	16.4 ± 2.6
Total development time (days)	42.7 ± 6.8	42.2 ± 5.4	41.8 ± 5.8
Sex ratio (male:female)	0.48	0.46	0.5
Fertility (eggs:adults)	0.67	0.68	0.75

Values shown are means ± SD of three independent experiments, asterisk denotes significant difference (P ≤ 0.05). For post-oviposition survival, numbers in parentheses represent percentage of flies surviving for at least 24 h. Numbers of insects used were 57 bloodfed on chickens, 54 bloodfed on dogs and 50 bloodfed on human, except for time to engorge where number of flies used are indicated.

Eggs were produced from 6 to 15 days after bloodfeeding, with no significant differences between the egg maturation times for females fed on any of the hosts (Table 1). Sand flies fed on chickens and dogs produced similar average numbers of eggs (64.3), however, both yielded significantly more eggs than those fed on human blood (48). This data indicates that in terms of immediate reproductive success human hosts are not a particularly good source of blood, but that chickens are as good as dogs. The human blood feeds led to significantly lower amounts protein ingested. In addition, human blood is deficient in isoleucine and this may also explain the relatively poor egg production [28]. Harrington et al. [29] found that *Aedes aegypti *had become adapted to feeding on isoleucine-poor human blood and was able to dispense with sugar-feeding when it was allowed to bite man. This adaptation resulted in mosquitoes taking smaller but more frequent blood meals and selected for anthropophilic behaviour, enhancing its efficiency as a vector of human-borne pathogens. This situation seems to be different with *Lu. longipalpis *where previous studies showed that this sand fly species is an opportunistic feeder and is not highly anthropophilic nor strongly attracted to dogs [30].

The longevity and post oviposition time survival times did not differ significantly after feeding on chicken, dog or human blood. The bloodmeal source did not have any influence on larval development. Eggs hatched 12-21 days after being laid, with mean values from 16.2 to 16.4 days (Table 1). Total development time from oviposition to adult emergence was 33-47 days, mean values ranging from 41.8 to 42.7 days. There were no significant differences in the proportions of male and female sand flies among the progeny of females fed on any of the three hosts (range 0.46 - 0.50). The numbers of adults produced as a proportion of eggs laid varied considerably within all three groups, but no significant differences were found between any of them. The mean proportions of progeny completing development from females that had fed on chicken, dog or human blood were 0.67, 0.68 and 0.75 respectively (Table 1).

Harre et al. [31] compared fecundity and survival rates among sand flies (*P. papatasi*) membrane fed on blood from eight species (including man) but found no significant differences between any of them. However, Noguera et al. [32] studying the reproductive potential of *Lu. ovallesi *found that chicken blood was more nutritious than that of several other species (goat, cow, pig, human, dog and horse), being digested more quickly and leading to the production of greater numbers of eggs.

### Sand fly infection via chicken blood

*Lutzomyia longipalpis *is the natural vector of the medically important parasite, *L. infantum*. The domestic chicken, *Gallus gallus*, has been implicated as an important maintenance host for the sand fly vector and bird ownership has been identified as a key risk factor for disease [33]. However, chickens are also known to resist *Leishmania *infection and the precise effect of chicken blood upon parasite development in the vector and subsequent transmission is unclear.

**Figure 3:**
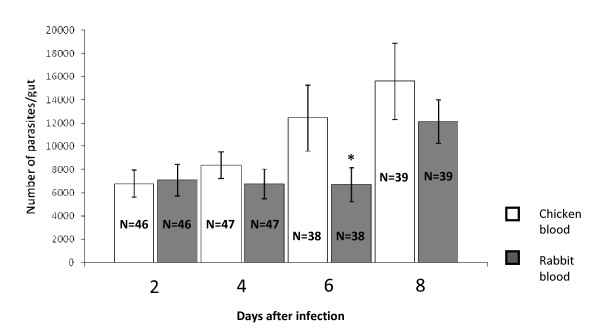
**Number of parasites in the midgut of sand flies infected with chicken and rabbit blood**. Mean number of parasites per sand fly gut at 2 day intervals post infection via rabbit or chicken blood (5 independent experiments). * represents statistical significance between chicken fed and rabbit fed at 6 days, P ≤ 0.0138 (*U *Mann-Whitney).

Comparison of *Leishmania *infection of sand flies via chicken or rabbit blood suggested that chicken blood was a good medium for establishing infections of *L. mexicana *in *Lu. longipalpis*. Feeding an amastigote infected chicken bloodmeal produced a consistently high (from 82.1 to 95.7%) percentage infection among the sand flies in comparison to the percentage infected amongst the rabbit blood fed group up to 6 days post-infection (Table 2). There was a trend towards higher numbers of parasites observed in sand flies given an infection via chicken blood compared to rabbit blood fed sand flies (Fig. 3), and 6 days after infection the number of parasites found in sand flies infected with chicken blood was significantly higher than in flies infected with rabbit blood (*U *Mann-Whitney, P ≤ 0.0138). This data is in accordance with the observations of Nieves and Pimenta [34] in *Lutzomyia migonei*. A higher parasite count in sand flies infected via chicken blood is of particular interest as the number of parasites is important to the transmission potential, for example contributing to the construction of the PSG plug [35] and number of infective forms.

**Table 2:** Effect of blood source on the percentage of *Lu. longipalpis *infected with *L. mexicana*.

Blood Source	% Flies infected (N° flies examined)			
	2 days	4 days	6 days	8 days
Rabbit blood	82.6	74.5	68.4	89.7
	(46)	(47)	(38)	(39)
				
Chicken blood	95.7	89.4	92.1	82.1
	(46)	(47)	(38)	(39)

### Effect of blood type on promastigote differentiation of *L. mexicana *in *Lu. longipalpis*

**Figure 4:**
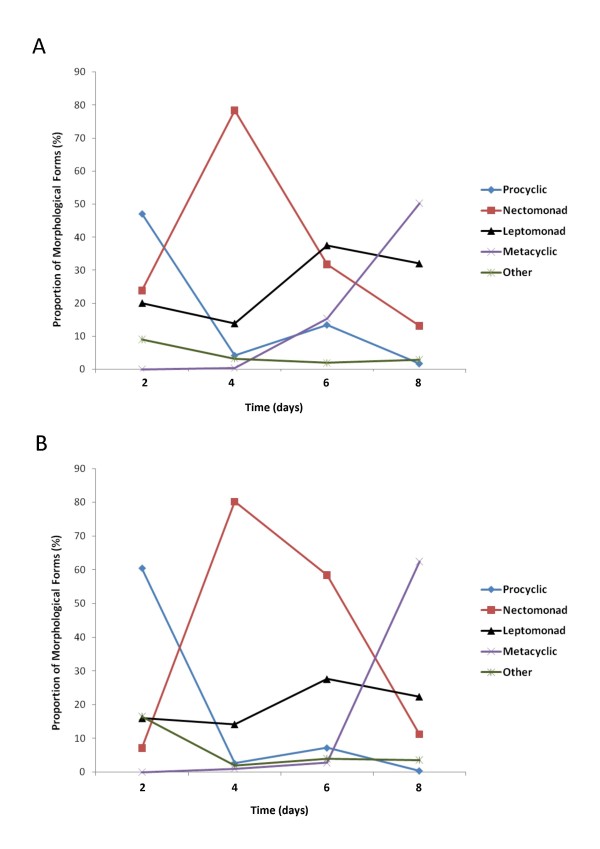
**Proportion of *Leishmania *morphological stages in the midgut of sand flies infected with chicken and rabbit blood**. Proportion of *Leishmania mexicana *morphological stages present every 48 hours for 8 days post-infection given with rabbit blood (A) and chicken blood (B). Data showed represents 2 independent pair-wise biological replicates.

As the midgut infection progresses, there are changes in promastigote morphology leading to the mammalian infective metacyclic form [18]. The relative proportion of different morphological *Leishmania *populations followed a similar pattern in both mammalian and avian blood (Fig. 4). The initial population was mainly identified as procyclic promastigotes followed by an increase in proportion of the nectomonad and leptomonad populations. This was followed by the appearance of the mammalian infective metacyclic form in both groups.

The frequencies of the different morphologies were significantly different between the parasite populations from sand-flies infected via rabbit and chicken blood (Fig. 4) at both 2 days (χ^2^(3) = 54.30, *p *≤ 0.01) and 6 days post infection (χ^2^(4) = 34.714, *p *≤ 0.01). After 2 days the parasites administered via chicken blood had a significantly higher number of procyclic parasites and lower frequency of nectomonads relative to the parasites from rabbit blood fed sand-flies. After 6 days, sand flies infected via chicken blood had significantly higher frequencies of nectomonads and leptomonads compared to flies infected by rabbit blood which had relatively more metacyclics. However, there was no significant effect of blood source on the frequencies of the different morphological forms at 4 days (χ^2^(4) = 3.958, *p *> 0.05) and 8 days post infection (χ^2^(4) = 8.617, *p *> 0.05) with both groups supporting predominantly metacyclic populations by day 8.

Overall, infection via chicken blood may be associated with a slightly slower developmental rate as these flies produced metacyclics later but parasites developed to the mammalian infective stage in similar proportions from both blood sources by the 8th day post infection. The results show the potential for *Lu. longipalpis *to produce a large population of mammalian infective metacyclic promastigotes in chicken blood. This contrasts with the work of Schlein et al. [6] where *Leishmania major *infection was inhibited in *Phlebotomus papatasi *after ingestion from avian turkey blood. The establishment of infection via chicken blood suggests that there is nothing within the chicken's circulating blood which is preventing infection, and therefore chickens do not permit *Leishmania *development for a different reason, such as their high body temperature as postulated by Hayatee et al. [36].

Infection via avian and mammalian blood sources progressed in a similar manner, concurring with the developmental profiles determined by Rogers et al. [18], though the time taken to reach the peak of each morphological form appeared to be longer in our experiments. This is most likely due to the lower ambient temperature of 24°C at which these infections were conducted in comparison to the previous study. Although the morphological counts detected a similar proportion of metacyclic forms present in flies infected via both whole chicken and rabbit blood by day 8 this translates into an absolute higher number of metacyclics in the chicken blood fed sand flies as they have a higher total *Leishmania *population. The possibility that this might lead to higher potential for transmission cannot be discounted.

In ZVL-endemic areas where a wide range of domestic animals are present, sand flies may often acquire partial bloodmeals from multiple host species. Although birds do not acquire a *Leishmania *infection, it is plausible that an avian bloodmeal could follow a *Leishmania *infected mammalian bloodmeal. The present study shows that chicken blood does not inhibit the development of *Leishmania *parasites in sand flies and that chickens are unlikely to offer any protection from disease but may, on the contrary, promote parasite growth and development in the vector thus increasing transmission potential. This information contributes to the growing evidence that chickens are not simply neutral in *Leishmania *transmission [2], and provide a partial explanation for the field observations of Moreno et al. [33], which exposed chicken ownership as a major risk factor for leishmaniasis.

The observation that chicken blood promotes parasite growth and development has important implications for vector control because the higher level of infection associated with chicken blood would potentially reduce any beneficial zooprophylactic effects of chickens [2]. There are limited options for health authorities in endemic areas of intense *Leishmania *transmission and banning chicken-rearing from urban environments is an unrealistic option. Potential alternatives are the removal of chicken coops from near human dwellings, treating chickens and coops with insecticides or the use of insecticide treated pheromone traps [11].

## Conclusions

It was concluded that chickens are suitable hosts to support a *Lu. longipalpis *population and that chicken blood is likely to support the development of transmissible *Leishmania *infections in *Lu. longipalpis*. The study provides information of direct benefit to public health authorities in Brazil that will allow them to potentiate existing VL control strategies, perhaps by limiting chicken-rearing in areas where the disease already exists or reducing the amount of residual insecticide spray as a result of improved targeting of spray sites.

## Competing interests

The authors declare that they have no competing interests.

## Authors' contributions

BA, PAB and RJD designed the study. MRVS, ED, AN, HMDA and RRC performed the experimental work. MRVS, ED, AN, BA and RJD analysed the data. MRVS, ED, BA, PAB and RJD prepared the manuscript, which was read and approved by all authors.

## Supplementary Material

Additional file 1 **Table A**. Analysis of chicken, rabbit, human and dog blood used in the experiments. Click here for file

Additional file 2 **Figure A**. Longevity of female sand flies after meal of 70% sucrose, chicken or rabbit blood. Click here for file

## References

[B1] Lainson R Rangel EF *Lutzomyia longipalpis *and the eco-epidemiology of American visceral leishmaniasis, with particular reference to Brazil: a review Mem Inst Oswaldo Cruz 2005 100 811 827 16444411 10.1590/s0074-02762005000800001

[B2] Alexander B de Carvalho RL McCallum H Pereira MH Role of the domestic chicken (*Gallus gallus*) in the epidemiology of urban visceral leishmaniasis in Brazil Em Infect Dis 2002 8 1480 1485 10.3201/eid0812.010485 PMC2738513 12498667

[B3] Michalsky EM Rocha MF da Rocha Lima AC França-Silva JC Pires MQ Oliveira FS Pacheco RS Dos Santos SL Barata RA Romanha AJ Fortes-Dias CL Dias ES Infectivity of seropositive dogs, showing different clinical forms of leishmaniasis, to *Lutzomyia longipalpis *phlebotomine sand flies Vet Parasitol 2007 147 67 76 10.1016/j.vetpar.2007.03.004 17449184

[B4] Ready PD Factors affecting egg production of laboratory-bred *Lutzomyia longipalpis *(Diptera: Psychodidae) J Med Entomol 1979 16 413 423 541814 10.1093/jmedent/16.5.413

[B5] Alder S *Leishmania* Adv Parasitol 1964 2 35 91 10.1016/S0065-308X(08)60586-2 14321783

[B6] Schlein Y Warburg A Schur LF Shomai J Vector compatibility of *Phlebotomus papatasti *on differentially induced digestion Acta Trop 1983 40 65 70 6134455

[B7] Corredor A Gallego JF Tesh RB Morales A De Carrasquilla CF Young DG Kreutzer RD Boshell J Palau MT Caceres E Epidemiology of visceral leishmaniasis in Colombia Am J Trop Med Hyg 1989 40 480 486 2729506 10.4269/ajtmh.1989.40.480

[B8] Castellon AJ Domingos ED On the focus of kala-azar in the state of Roraima, Brazil Mem Inst Oswaldo Cruz 1991 86 375 10.1590/S0074-02761991000300014 1842427

[B9] Arias JR Monteiro PS Zucker F The re-emergence of visceral leishmaniasis in Brazil Em Infect Dis 1996 2 145 146 10.3201/eid0202.960213 PMC2639817 8903218

[B10] Caldas AJ Costa JM Silvam AA Vinhas V Barral A Risk factors associated with asymptomatic infection by *Leishmania chagasi *in north-east Brazil Trans R Soc Trop Med Hyg 2002 96 21 28 10.1016/S0035-9203(02)90227-0 11925984

[B11] Bray DP Bandi KK Brazil RP Oliveira AG Hamilton JGC Synthetic sex pheromone attracts the leishmaniasis vector *Lutzomyia longipalpis *(Diptera: Psychodidae) to Traps in the Field J Med Entomol 2009 46 428 434 10.1603/033.046.0303 19496409 PMC3197723

[B12] Lewis JH Comparative haemostasis in vertebrates 1996 Plenum Press, New York

[B13] Sádlová J Reishig J Volf P Prediuresis in female *Phlebotomus *sandflies (Diptera: Psychodidae) Eur J Entomol 1998 95 639 642

[B14] Souza AV Petretski JH Demasi M Bechara EJ Oliveira PL Urate protects a blood-sucking insect against hemin-induced oxidative stress Free Rad Biol Med 1997 22 209 214 10.1016/S0891-5849(96)00293-6 8958146

[B15] Sant'Anna MRV Alexander JB Bates PA Dillon RJ Gene silencing in phlebotomine sand flies: xanthine dehydrogenase knock down by dsRNA micro-injections Insect Biochem Mol Biol 2008 38 652 660 10.1016/j.ibmb.2008.03.012 18510977 PMC2677462

[B16] Modi GB Crampton JM, Beard CB, Louis C Care and maintenance of phlebotomine sandfly colonies The Molecular Biology of Insect Disease Vectors 1997 London, Chapman & Hall 21 30

[B17] Bradford MM A rapid and sensitive method for the quantitation of microgram quantities of protein utilizing the principle of protein-dye binding Anal Biochem 1976 72 248 254 10.1016/0003-2697(76)90527-3 942051

[B18] Rogers ME Chance ML Bates PA The role of promastigote secretory gel in the origin and transmission of the infective stage of *Leishmania mexicana *by the sandfly *Lutzomyia longipalpis* Parasitology 2002 124 495 507 10.1017/S0031182002001439 12049412

[B19] Bates PA Robertson CD Tetley L Coombs GH Axenic cultivation and characterization of *Leishmania mexicana *amastigote-like forms Leishmania mexicana 1992 105 193 202 10.1017/s0031182000074102 1454417

[B20] Bates PA Crampton JM, Beard CB, Louis C Infection of phlebotomine sandflies with *Leishmania* The Molecular Biology of Insect Disease Vectors: A methods manual 1997 London, Chapman & Hall 112 120

[B21] Freeman BM Flack IH Effects of metyrapone on plasma corticosterone concentration in *Gallus domesticus* Comp Biochem Physiol 1984 79 113 116 10.1016/0300-9629(84)90545-0 6149854

[B22] Briegel H Rezzonico L Concentration of host blood protein during feeding by anopheline mosquitoes (Diptera: Culicidae) J Med Entomol 1985 22 612 618 4078846 10.1093/jmedent/22.6.612

[B23] Shortt HE Swaminath CS The method of feeding of *Phlebotomus argentipes *with relation to its bearing on the transmission of Kala-Azar Indian J Med Research 1928 15 827 836

[B24] Sádlová J Volf P Occurrence of *Leishmania major *in sandfly urine Parasitology 1999 118 455 460 10.1017/S0031182099004254 10363278

[B25] Guarneri AA Pereira MH Diotaiuti L Influence of the blood meal source on the development of *Triatoma infestans, Triatoma brasiliensis, Triatoma sordida*, and *Triatoma pseudomaculata *(Heteroptera, Reduviidae) J Med Entomol 2000 37 373 379 10.1603/0022-2585(2000)037[0373:IOTBMS]2.0.CO;2 15535580

[B26] Glantzounis GK Tsimoyiannis EC Kappas AM Galaris DA Uric acid and oxidative stress Curr Pharm Des 2005 11 4145 4151 10.2174/138161205774913255 16375736

[B27] Hilliker AJ Duyf B Evans D Phillips JP Urate-null rosy mutants of D *rosophila melanogaster *are hypersensitive to oxygen stress Proc Natl Acad Sci USA 1992 89 4343 4347 10.1073/pnas.89.10.4343 1316606 PMC49078

[B28] Greenberg J Some nutritional requirements of adult mosquitoes (*Aedes aegypti*) for oviposition J Nutr 1951 43 27 35 14851026 10.1093/jn/43.1.27

[B29] Harrington LC Edman JD Scott TW Why do female *Aedes *aegypti (Diptera: Culicidae) feed preferentially and frequently on human blood? J Med Entomol 2001 38 411 422 10.1603/0022-2585-38.3.411 11372967

[B30] Morrison AC Ferro C Tesh RB Host preferences of the sand fly Lutzomyia longipalpis at an endemic focus of American visceral leishmaniasis in Colombia Am J Trop Med Hyg 1993 49 68 75 8352394 10.4269/ajtmh.1993.49.68

[B31] Harre JG Dorsey KM Armstrong KL Burge JR Kinnamon KE Comparative fecundity and survival rates of *Phlebotomus papatasi *sandflies membrane fed on blood from eight mammal species Med Vet Entomol 2001 15 189 196 10.1046/j.1365-2915.2001.00278.x 11434553

[B32] Noguera P Rondón M Nieves E Effect of blood source on the survival and fecundity of the sandfly *Lutzomyia ovallesi *Ortiz (Diptera: Psychodidae), vector of *Leishmania* Biomedica 2006 26 57 63 17361842

[B33] Moreno EC Melo MN Genaro O Lambertucci JR Serufo JC Andrade ASR Antunes CMF Carneiro M Risk factors for *Leishmania chagasi *infection in an urban area of Minas Gerais State Rev Soc Bras Med Trop 2005 38 456 463 16410918 10.1590/s0037-86822005000600002

[B34] Nieves E Pimenta PFP Influence of vertebrate blood meals on the development of *Leishmania (Viannia) braziliensis *and *Leishmania (Leishmania) amazonensis *in the sand fly *Lutzomyia migonei *(Diptera: Psychodidae) Am J Trop Med Hyg 2002 67 640 647 12518856 10.4269/ajtmh.2002.67.640

[B35] Rogers ME Ilg T Nikolaev AV Ferguson MAJ Bates PA Transmission of cutaneous leishmaniasis by sand flies is enhanced by regurgitation of fPPG Nature 2004 430 463 467 10.1038/nature02675 15269771 PMC2835460

[B36] Hayatee ZG Effect of environmental temperature on cutaneous leishmaniasis diffusa in mice Trans R Soc Trop Med 1970 64 679 682 10.1016/0035-9203(70)90005-2 5442055

