# Supplementary material for: Chicken blood provides a suitable meal for the sand fly Lutzomyia longipalpis and does not inhibit Leishmania development in the gut
Source: Parasit Vectors. 2010 Jan 11;3:3. doi: 10.1186/1756-3305-3-3 (PMC2823724; doi:10.1186/1756-3305-3-3)
Supplement: Additional file 1 — Table A. Analysis of chicken, rabbit, human and dog blood used in the experiments. [file 1756-3305-3-3-S1.DOC]

Additional file 1

**Table A. Analysis of chicken, rabbit, human and dog blood used in the experiments.**

|  | Chicken blood  (n=6) | Rabbit blood  (n=14) | Human blood  (n=5) | Dog blood  (n=3) |
| --- | --- | --- | --- | --- |
| WBC | ND | 2.77 ± 1.54 x 10^3^ | 5.42 ± 2.45 x 10^3^ | 7.27 ± 4.20 x 10^3^ |
| RBC | 1.11 ± 0.18 x 10^6^ | 2.95 ± 0.72 x 10^6^ | 3.64 ± 1.12 x 10^6^ | 6.08 ± 1.33 x 10^6^ |
| Protein | 89.10 ± 10.48* | 217.06 ± 27.21 | 236.97 ± 26.49 | 251.52 ± 26.12 |
| Hgb | 4.98 ± 0.96* | 6.12 ± 1.72** | 10.92 ± 2.79 | 14.87 ± 2.90 |
| Hct | 15.08 ± 2.56 | 21.42 ± 4.81 | 33.64 ± 9.23 | 43.50 ± 8.87 |
| MCV | 135.19 ± 3.73 | 73.00 ± 3.69 | 95.12 ± 4.11 | 71.70 ± 3.20 |
| MCH | 44.71 ± 3.22 | 20.68 ± 1.42 | 31.12 ± 1.27 | 24.57 ± 0.61 |
| MCHC | 33.06 ± 1.67 | 28.35 ± 1.90 | 32.74 ± 1.71 | 34.33 ± 1.25 |
| Platelets | **-** | 173.54 ± 81.21 x 10^3^ | 141.20 ± 81.13 x 10^3^ | 145.00 ± 13.23 x 10^3^ |

WBC - white blood cell count (number of cells/μL); RBC - red blood cell count (number of cells/μL); Protein – total blood protein concentration (mg/mL); Hgb – haemoglobin protein concentration (g/dL); Hct - hematocrit (% of total volume occupied by red blood cells); MCV - mean corpuscular volume (average size of red blood cells in femtoliters); MCH - mean corpuscular haemoglobin (amount of haemoglobin per red blood cell in pg); MCHC - mean corpuscular haemoglobin concentration (haemoglobin concentration inside red blood cells in g/dL); Platelets – platelet count (number of platelets/μL). Results are presented as mean ± SD. ND = not determined.*represents statistical significance at P≤0.024 between chicken blood and all other blood sources.**represents statistical significance at P≤0.006 between rabbit blood and human and dog blood (*U* Mann-Whitney).
