# Supplementary material for: Chicken blood provides a suitable meal for the sand fly Lutzomyia longipalpis and does not inhibit Leishmania development in the gut
Source: Parasit Vectors. 2010 Jan 11;3:3. doi: 10.1186/1756-3305-3-3 (PMC2823724; doi:10.1186/1756-3305-3-3)
Supplement: Additional file 2 — Figure A. Longevity of female sand flies after meal of 70% sucrose, chicken or rabbit blood. [file 1756-3305-3-3-S2.DOC]

**Additional File 2**

**Figure A. Longevity of female sand flies fed on sucrose, chicken or rabbit blood .**

Longevity of female *Lu longipalpis* after 70% sucrose feed (N=364) and blood feed on chicken (N=410) and rabbit blood (N=590). Log-rank (Mantel-Cox) test was performed (χ2: 0.6673; P=0.4140) between chicken and rabbit blood-fed sand flies. 3 independent experiments were performed.
